# Influencing walking behavior can increase the physical activity of patients with chronic pain hospitalized for multidisciplinary rehabilitation: an observational study

**DOI:** 10.1186/s12891-019-2561-9

**Published:** 2019-05-04

**Authors:** Philippe Terrier, Caroline Praz, Joane Le Carré, Philippe Vuistiner, Bertrand Léger, François Luthi

**Affiliations:** 1 Haute Ecole Arc Santé, HES-SO University of Applied Sciences and Arts Western Switzerland, Espace de l’Europe 11, 2000 Neuchâtel, Switzerland; 2 IRR, Institute for Research in Rehabilitation, Sion, Switzerland; 3 Clinique romande de réadaptation Suva, Sion, Switzerland; 4 Department of Physical Medicine and Rehabilitation, Orthopaedic Hospital, Lausanne University Hospital, Lausanne, Switzerland

**Keywords:** Orthopedic trauma, Chronic musculoskeletal pain, Multidisciplinary biopsychosocial rehabilitation, Accelerometer, Physical functioning, Pain interference

## Abstract

**Background:**

Physical therapy and exercising are key components of biopsychosocial rehabilitation for chronic pain. Exercise helps reduce pain and improve physical functions. In addition, a high level of physical activity benefits quality of life and emotional well-being. However, the degree to which hospitalization for extensive rehabilitation effectively increases physical activity has not yet been studied. Therefore, we investigated the physical activity level and the walking behavior of inpatients with musculoskeletal pain. The objectives were 1) to compare physical activity level and walking with or without rehabilitation, 2) to evaluate whether pain site influences physical activity level, and 3) to measure the association between physical activity and pain-related interference with physical functioning.

**Methods:**

During a rehabilitation stay, 272 inpatients with lower limb, spine, or upper limb pain wore an accelerometer over 1 week. We assessed the daily duration of the practice of moderate physical activity and walking. Weekend days, during which the participants went home (days off), were used as a reference for habitual activities. We also evaluated 93 patients before the hospitalization to validate the use of days off as a baseline. Pain interference was measured with the brief pain inventory questionnaire. Generalized linear mixed models analyzed the association between physical activity and walking levels, and 1) rehabilitation participation, 2) pain sites, and 3) pain interference.

**Results:**

Weekend days during the stay have similar physical activity level as days measured before the stay (73 min / day at the clinic, versus 70 min / day at home). Rehabilitation days had significantly higher physical activity levels and walking durations than days off (+ 28 min [+ 37%] and + 32 min [+ 74%], respectively). Mixed models revealed 1) a negative association between physical activity and pain interference, and 2) no effect of pain sites. Overall, patients increased their physical activity level independently of reported pain interference.

**Conclusions:**

Despite their painful condition, the inpatients were able to engage themselves in a higher level of physical activity via increased participation in walking activities. We conclude that walking incentives can be a valid solution to help patients with chronic pain be more physically active.

**Electronic supplementary material:**

The online version of this article (10.1186/s12891-019-2561-9) contains supplementary material, which is available to authorized users.

## Background

A high prevalence of chronic pain—around 20% of the adult population—is observed all over the world [1–3]. This debilitating condition, which is particularly difficult to treat, causes a substantial burden to healthcare systems, economies, and societies [4]. Due to its high prevalence, complex etiology, and the need for combined therapeutic approaches, chronic pain constitutes a major challenge for practitioners, caregivers, and researchers.

Traumatic injuries and orthopedic traumas are frequent causes of chronic pain. Six month after an isolated musculoskeletal extremity injury (fracture, sprain, or strain), 10% of patients suffer chronic pain [5]. The mechanisms that explain the transition from acute to chronic pain following a trauma are still incompletely understood [6]. That said, psychological factors may explain the continuation of disability and pain after a skeletal trauma [7]. Following an orthopedic trauma, strategies that combine physical and psychological therapies may thus help prevent the occurrence and continuation of chronic pain [8].

Multidisciplinary biopsychosocial rehabilitation is the favored approach to severe chronic pain that is resistant to other treatments [9–11]. Although analgesic medication is indeed used as a primary treatment, poor outcomes are often observed [12–14]. Psychological and social approaches are thus required, along with biomedical care and physical therapy, to enhance patient’s ability to function with pain through coping strategies [15, 16]. Multidisciplinary rehabilitation is particularly beneficial to patients with poor prognosis when therapies are combined at high frequency, e.g., six-hour sessions 5 days per week over 4 weeks [17].

Exercise and physical therapy are key components of biopsychosocial rehabilitation [9, 18]. Exercise aims to improve cardiovascular fitness (aerobic training [19]), increase joint mobility and reduce muscle stiffness (flexibility training [20]), and enhance muscle strength (strength or resistance training [20, 21]). Exercise in general [18, 22, 23], and walking in particular [24], improve function and reduce pain. Furthermore, the WHO recommendations on physical activity and health [25] advise participation in at least 150 min per week of moderate-to-vigorous physical activity (MVPA). Indeed, a positive association exists between physical activity, health-related quality of life [26], and emotional well-being [27]. Evidence also exists that being physically active has positive effects on painful conditions [28]. Therefore, encouraging patients to walk more and to be more physically active is a desirable goal of chronic pain care. In addition to specific effects on functional capacity, physical therapy may increase the level of physical activity, which can further improve a patient’s well-being. However, the immediate effects of intense multidisciplinary rehabilitation on daily physical activity and walking behavior have not been studied, especially during hospitalization.

Knowing how patients perceive their own functioning is essential to the study of chronic pain in general [29, 30], and pain-activity associations in particular. Psychological distress and fear [31], as well as beliefs, coping and catastrophizing [32], and self-efficacy [33], also play a role in how chronic pain impairs patients’ functioning. Measuring the self-perceived interference of pain with the practice of daily-life activities is thus recommended [29].

We were interested in the immediate effects that a rehabilitation stay may have on the physical activity levels of patients suffering from chronic pain following an orthopedic trauma. We aimed at investigating the difference between habitual physical activity and physical activity during rehabilitation. Furthermore, we sought to assess the proportion of total physical activity due to walking activities (WA), and to what extent this proportion was modified during the hospitalization. Finally, we sought to determine whether pain interference with physical functioning, as well as pain localization, could modulate physical activity levels.

## Methods

### Study design and setting

This study is a single-center, cross-sectional study that analyzed the physical activity level of patients with musculoskeletal pain during hospitalization for rehabilitation. The study is part of a larger ongoing cohort study aimed at a better understanding of the relationships between pain-related behaviors, functional deficits, and rehabilitation outcomes.

Between October 2013 and October 2017, we screened patients admitted for multidisciplinary biopsychosocial rehabilitation in the department of musculoskeletal rehabilitation of the Clinique Romande de Réadaptation (Sion, Switzerland). With a capacity of 95 beds, the department rehabilitates patients with moderate to severe after-effects following an orthopedic trauma. After two to three days of medical evaluation through examination, functional testing, interviews, and questionnaires, inpatients follow an interdisciplinary program for three to five hours, five days a week for five weeks. The program includes physical rehabilitation, exercise therapy, vocational rehabilitation, and psychological support (cognitive-behavioral therapy). For more information about the rehabilitation program, refer to our recent article [34].

### Participants

Study candidates had functional impairments and were unable to return to work after orthopedic trauma following work, traffic, sport, or leisure accidents. The eligibility criteria were as follows: 1) chronic pain > 3 months, 2) age > 18 years and < 65 years, 3) no amputation, 4) walking without aids, 5) no severe comorbidities, 6) French speaking, 4) live in Switzerland. We further excluded eligible patients who refused to participate in the cohort study and eligible patients who agreed to participate in the cohort study but specifically declined to wear the accelerometer.

We classified the patients into four categories according to their injury sites: 1) lower limbs (LoL), including participants with injuries to the foot & ankle, knee, hip, shank or thigh; 2) spine (Sp), including participants with cervical, thoracic, or lumbar spine injuries; 3) upper limbs (UpL), including participants injured at the shoulder, elbow, wrist, hand, arm, or forearm; and 4) polytrauma patients, including patients with more than one injury site.

Data on patient age, sex, body mass and height were collected. We used three questions from the brief pain inventory questionnaire (BPI interference scales [29, 35]) to assess physical functioning (i.e., pain-related interference with activities, hereafter referred to as pain interference [PI]). Specifically, we averaged the scores (0–10) of the following items: “how, during the last week, has pain interfered with your 1) general activities; 2) walking activities; 3) normal work (includes both work outside the home and housework).”

### Bias

Due to insurance particularities, the patients at our clinic are mostly blue-collar workers [36]. Therefore, women were underrepresented in the study’s sample. Another selection bias was that included patients might not adequately represent the population of hospitalized patients in our clinic. In particular, given the inclusion criteria, we suspected that complex cases would be underrepresented [37]. To assess this bias, we used the INTERMED score [38], which is routinely employed to assess the biopsychosocial complexity of hospitalized patients at our clinic [36]. The INTERMED is an observer-rated instrument that summarizes information from, biological, psychological, social, and health care domains. The INTERMED scores range from 0 to 60. Patients with scores beyond 20 are considered as complex cases [36].

### Instrument

The Actigraph wGT3X-BT activity monitor (Actigraph, Pensacola, FL, USA) recorded body accelerations of the participants [39]. This small and lightweight sensor (19 g), equipped with 4GB memory, was set to record 3-D accelerations (±8 g) at 50 Hz over 1 week. The device was attached to the right hip with an elastic belt, which is an optimal placement to assess physical activity [40].

### Procedure

We asked the participants to wear the accelerometer during waking hours, from awakening to bedtime. The participants removed the activity tracker when they performed aquatic activities. The assessment of physical activity took place during the second week of their stay, from Friday to the following Thursday (seven consecutive days). The participants went home for the weekend (days off) from Friday evening to Sunday evening.

In order to validate the use of days-off activity level as a proxy for habitual physical activity, we also investigated the physical activity level of some participants at home before the rehabilitation stay. This convenience subsample was chosen based on organizational criteria: the included patients had to know their hospitalization date 2 weeks in advance. They received the accelerometer by mail and wore it over the course of 1 week, 1 week before the hospitalization. The device was set to record acceleration from Wednesday to the next Tuesday. They returned the device at their arrival at the clinic.

### Data processing

Data processing is detailed in a previous paper [41]. In short, the one-week signals were sorted into daily signals. The days were tagged as week days (Monday–Friday) or weekend days (Saturday–Sunday). Days with less than 10 h of recording were discarded [42]. Next, we converted the 3D-signals into vector norms. We computed the signal amplitude every second (root mean square, RMS). We applied a cut-off at 0.1 g RMS for partitioning moderate-to-vigorous physical activity (MVPA) from sedentary-to-light physical activity. The 0.1 g threshold was empirically determined from the RMS values measured during walking [41]. In parallel, we specifically detected walking bouts with a custom algorithm based on the intensity and the dominant frequency of the acceleration signal [41].

To characterize physical activity level and walking behavior, we used a cascading scheme that divided total daily activity into subcomponents. We used four activity components for each recorded day: 1) the total wear time (hour / day), which is the time during which the subjects wore the accelerometer; 2) the MVPA, which is the time (minutes / day) spent performing activities of moderate-to-high intensity; 3) the walking activity (WA), which is the time spent walking during the day (minutes / day); 4) the long walks (LW), which is the cumulative time (minutes / day) of long walking bouts (> 1 min). Note that we did not normalize the duration of activity components by 24 h or wear time in order to obtain a total daily activity outcome and not an activity rate.

### Statistics

We utilized days as observational units to maximize the use of available data. In other words, we did not average the daily results across participants. The dependent continuous variables were MVPA, WA and LW, as observed for each available day. The main categorical independent variable was the day type (i.e., rehabilitation days or days off). In the subsample of subjects who wore the accelerometer at home before the stay, we defined 4 day types as follows: 1) home weekend; 2) home week; 3) clinic weekend; and 4) clinic week. Injury site (LoL, Sp, UpL) was considered as a categorical covariate. The categorical variables were converted into dummy variables. After centering, the pain interference (PI, 0–10) was included as a continuous covariate.

Distribution plots (violin plots) were used to show the distribution of the dependent variables. Because skewed distributions were expected, we reported medians, interquartile ranges (IQR), and quartiles (Q25 and Q75) to summarize the results.

For statistical inference, we applied hierarchical models with days nested in participants. See the recent study by Murphy et al. [43] for an example of hierarchical modeling in the field of physical activity and chronic pain. We computed generalized linear mixed models (hereafter: mixed models) given the right-skewed nature of the results (see Additional file 1: Figure S1–Figure S4). We used the Gamma distribution with the log-link function, as recommended by others for modeling right-skewed lengths of stay [44]. The subjects—coded with their unique ID number—were considered as a random effect. We set a random intercept and a random slope (subject x day type).

For multivariable models, the interactions between predictors were first examined through analyses of variance. Only the significant interactions were kept in the final models. To better visualize interactions, model outputs were illustrated with scatter plots of predicted marginal (fixed) effects. The threshold for statistical significance was set to 0.05. Complete results of the mixed models are published in Additional file 1: Tables S1-S10, along with the scatter plots of residuals (Additional file 1: Figure S5).

According to our goals, we built seven mixed models as follows:

- Model 1: Potential differences between days spent at home and at the clinic in the subsample of participants. Univariable model that included MVPA as the dependent variable and the day type as the independent variable.
- Models 2–4: Potential differences between rehabilitation days and days off in the whole sample. Univariable models that included MVPA (model 2), WA (model 3) and LW (model 4) as the dependent variables and the day type as the independent variable.
- Models 5–7: Determinants of physical activity level and walking behavior. Multivariable models that included MVPA (model 5), WA (model 6), and LW (model 7) as the dependent variables and day type, PI, and injury site as the independent variables.

### Sample size

Following the empirical rule of 15 cases per model parameter [45], we foresaw that 180 participants would be the minimum needed for the study’s statistics. This was based on the fact that the most complex model had 12 parameters (including interactions). However, this was a very rough estimation, given the hierarchical nature of our data (days nested within participants). We sought to include a larger number of participants to ensure that we had enough daily observations to accurately model within- and between-subjects variance. Therefore, the target of included participants was 300.

## Results

### Participants’ enrollment and characteristics

**Fig. 1:**
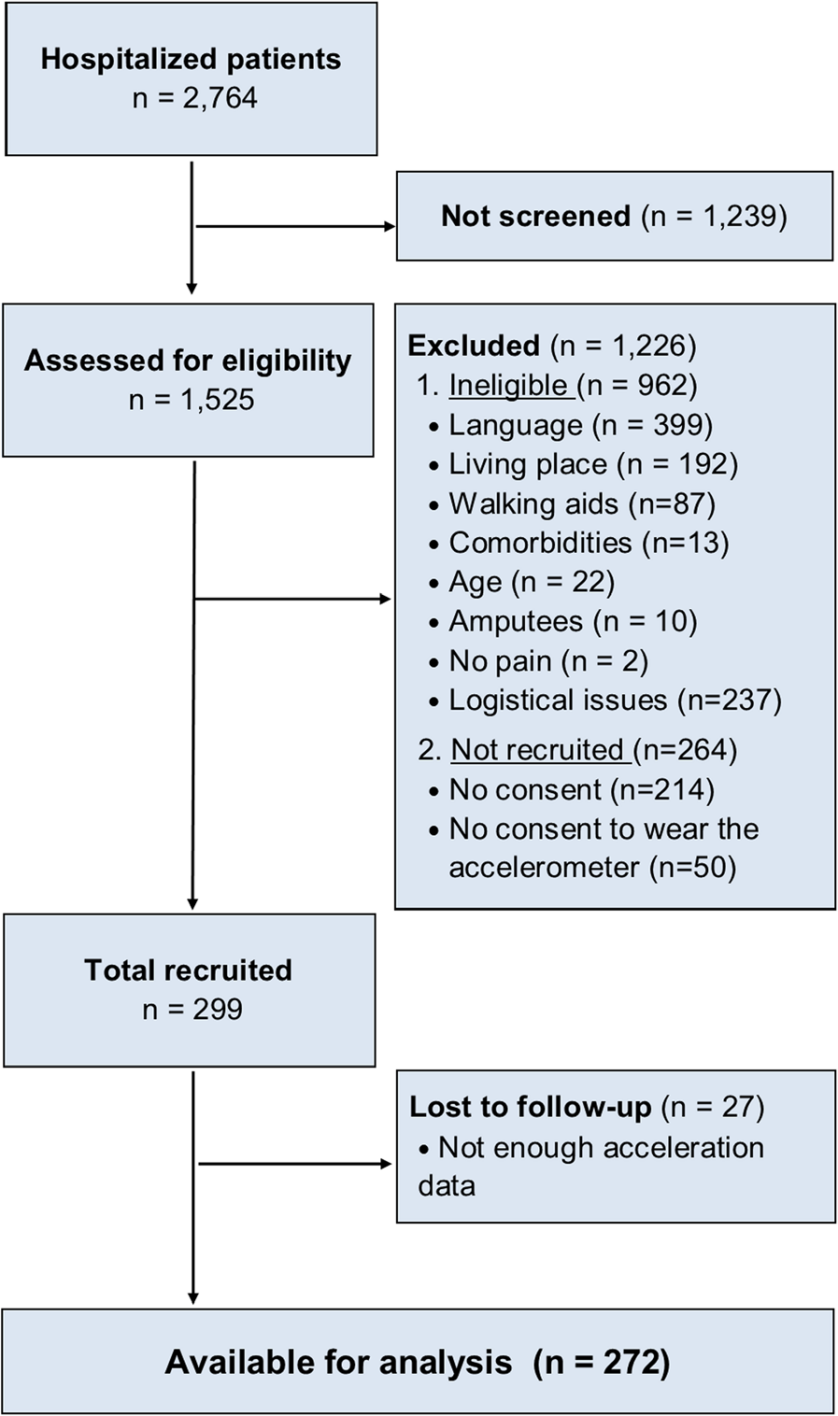
Flow diagram of study participants

The patients’ enrollment flow chart is shown in Fig. 1. We obtained data for 272 patients, from which 93 wore the accelerometer at home before hospitalization (Table 1). As expected, women were underrepresented (21%). The mean age was 44 years. Patients with upper-limb pain were the most represented group (42%), followed by lower limb pain (33%), and back pain (22%). Given the small number of patients with polytrauma (2%), we excluded this category from the models that explored the effects of injury site and PI (models 5–7). Regarding PI, we experienced one missing value, which was imputed with the mean value.

**Table 1:** Participants’ characteristics

	Sample 1		Sample 2	
	n	Mean (SD) or %	n	Mean (SD) or %
Age (years)	93	45 (11)	272	44 (12)
Sex				
Male	76	82%	214	79%
Female	17	18%	58	21%
Body mass (kg)	93	82 (16)	272	81 (17)
Body height (cm)	92	172 (8)	269	172 (8)
Pain interference [0–10]	92	5.6 (2.3)	271	5.3 (2.2)
Injury site				
Lower limbs	31	33%	91	33%
Spine	18	19%	61	22%
Upper limbs	43	46%	115	42%
Polytrauma	1	1%	5	2%
INTERMED [0–60]	93	22.0 (5.6)	272	21.9 (5.9)

Sample 1: Subsample of the participants who also wore the accelerometer at home before the rehabilitation stay. Sample 2: Full sample of all the participants measured during the rehabilitation stay

The average biopsychosocial complexity of the participants was not different from the typical complexity of inpatients of the Clinique Romande de Réadaptation. Among the 4997 patients hospitalized at the department of musculoskeletal rehabilitation between 2013 and 2017, the average INTERMED score was 22.1 (6.7), which represents a non-significant difference of 0.2 in comparison with the study’s sample (t-test with equal variance two-sided *p* = .61).

### Model 1: differences between days at home before the stay and at the clinic

In the subsample of 93 patients who were also assessed before the stay, we measured on average 9.2 days per subject. We obtained 1.4 days for the weekend at home, 3.7 days for the week at home, 1.1 day for the weekend at the clinic and 3.0 days for the week at the clinic.

**Fig. 2:**
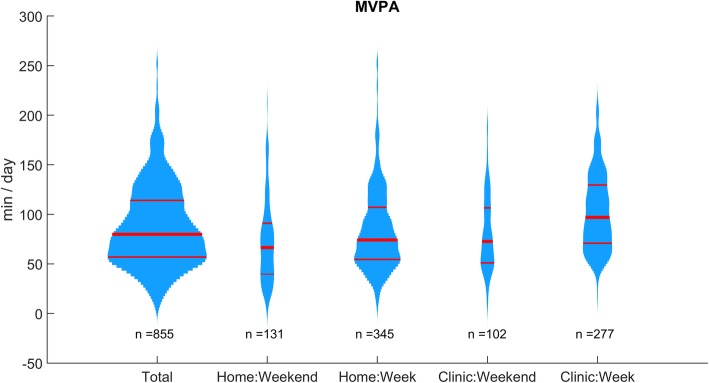
Distribution plots of the daily moderate-to-vigorous physical activity (MVPA) at home before the stay and at the clinic (93 subjects). Red lines show medians and quartiles. The width is proportional to the number of observations

As expected, the distributions of MVPAs were right-skewed (Fig. 2 and Additional file 1: Figure S2). At first sight, the patients were more active during the rehabilitation days. The medians shown in Table 2 highlight that patients indeed spent 30 more minutes performing MVPA during the hospitalization than during the weekend at home. MVPA times during the weekend at the clinic and the week at home were very close (one-minute difference).

**Table 2:** MVPA at home before the stay and at the clinic

*n* = 93 subjects		Weekend home	Week home	Weekend clinic	Week clinic
MVPA (min / day)	Q25	40	55	51	71
	Median	67	74	73	97
	Q75	91	107	107	130

*MVPA* Moderate-to-vigorous physical activity, *Q25* first quartile, *Q75* third quartile

The mixed models (Model 1, Table 3) inferred that a significant difference existed between day types. Model coefficients revealed that patients were 48% more active during rehabilitation days than during the weekend spent at home before the stay. Weekdays at home and weekend days at the clinic showed similar differences in comparison to weekend days at home (difference of 17 and 13%, respectively).

**Table 3:** MVPA differences between day types

Model 1	Coefficient	Confidence interval		% change	Confidence interval	
(*n* = 855 days)	Estimate	Lower	Upper	*exp*(coeff.)-1	Lower	Upper
(Intercept)	**4.174**	4.060	4.288	(65.0)	(58.0)	(72.8)
Day type						
(Weekend home)						
Week home	**0.155**	0.079	0.232	17%	8%	26%
Weekend clinic	**0.124**	0.030	0.219	13%	3%	24%
Week clinic	**0.388**	0.273	0.504	48%	31%	66%

Fixed effects of generalized linear mixed model (Gamma distribution with log link). Full model output is shown in Additional file 1: Table S1. Boldface indicates that the coefficient is significantly different from zero. *MVPA* moderate-to-vigorous activity

### Model 2–4: differences between rehabilitation days (week) and days off (weekend)

In the entire sample of 272 participants, we measured 5.1 days per subject divided into 3.7 rehabilitation days and 1.4 days off.

**Fig. 3:**
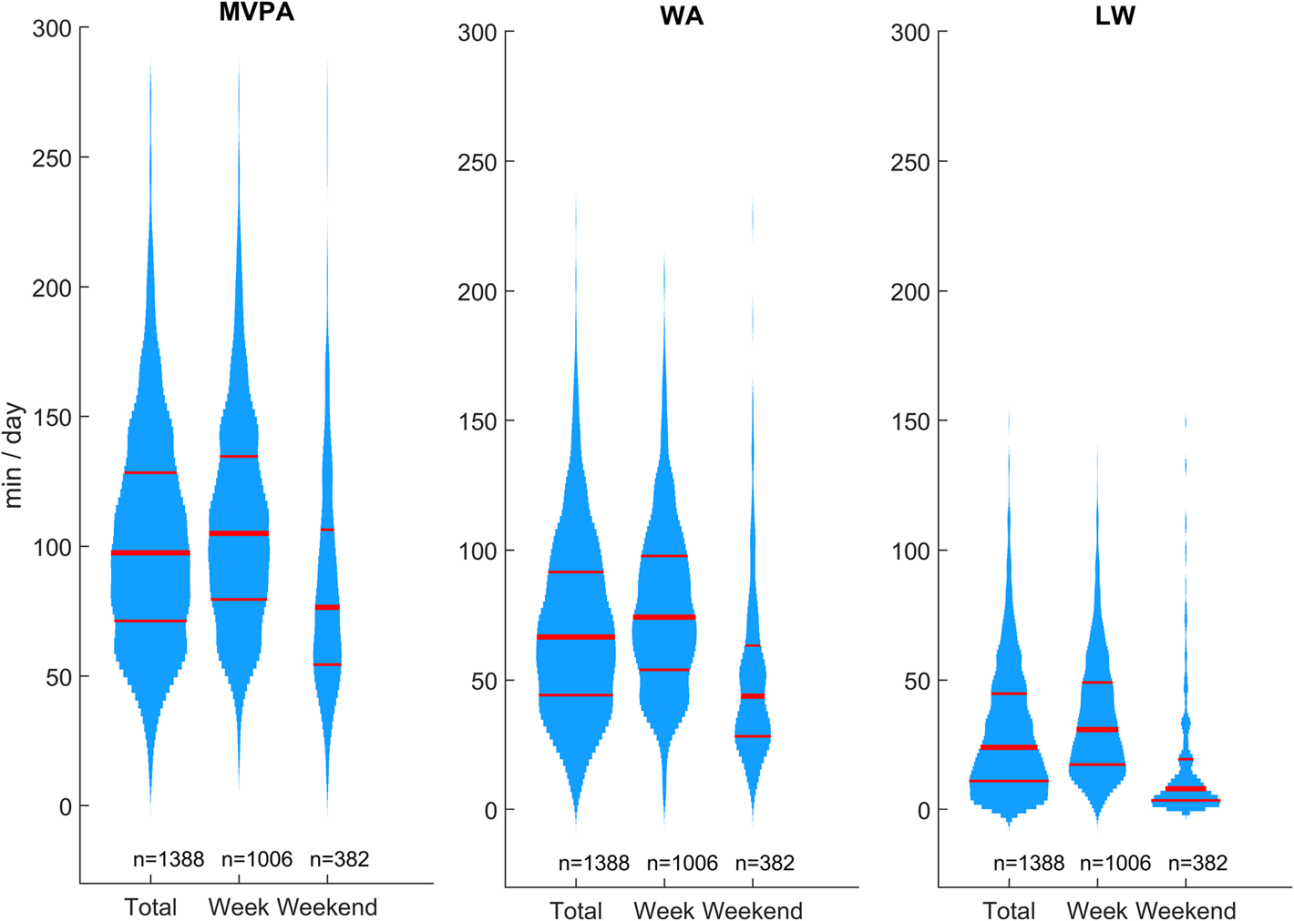
Distribution plots of the daily moderate-to-vigorous physical activity (MVPA), walking activity (WA) and long walk (LW) duration during the rehabilitation stay by day types (272 subjects). Week: rehabilitation days. Weekend: days off. Red lines show medians and quartiles. The width is proportional to the number of observations

Figure 3 shows the distributions of the activity variables. LW exhibited a strong right-skewness, particularly on weekend days. Although WA and MVPA are less skewed, the Gamma distribution fit the data better than the Gaussian distribution did (see Additional file 1: Figure S1-S4).

**Fig. 4:**
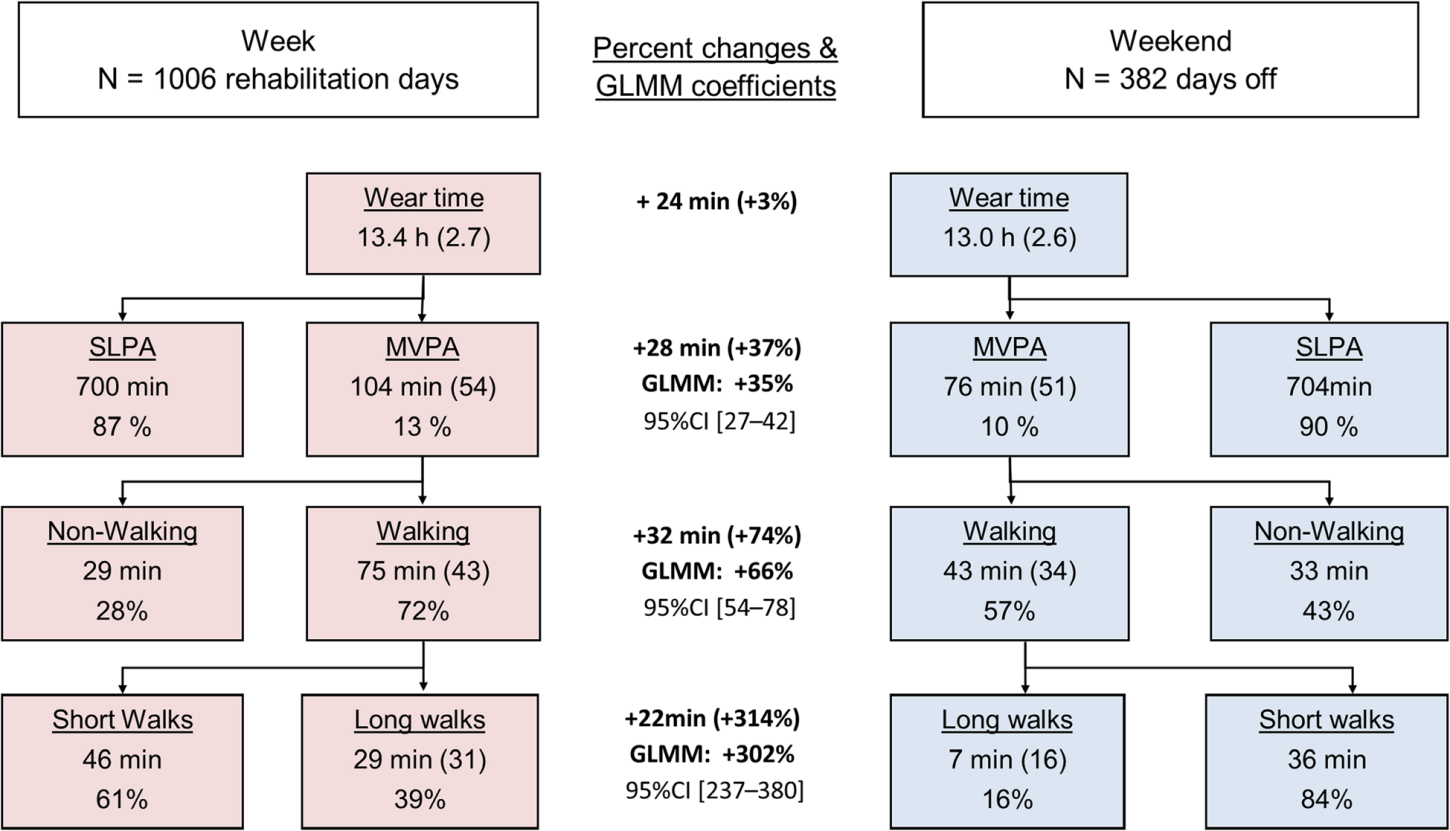
Repartition of physical and walking activity split by day types. SLPA: sedentary-to-low physical activity. MVPA: moderate-to-vigorous physical activity. GLMM: generalized linear mixed model

Figure 4 summarizes the differences between both day types. During days off, the subjects performed 7 min of long duration walks versus 29 min during the rehabilitation days. The subjects also performed more walking activities at the clinic (75 min versus 43 min) and were more physically active (104 min versus 76 min). The percent change between days was computed from the medians and inferred from the mixed models (full model outputs are shown in Additional file 1: Tables S2-S4). Although the mixed models tend to be slightly more conservative, both approaches confirm a substantial effect of the rehabilitation stay on MVPA, WA, and LW.

### Model 5–7: determinants of MVPA, WA, and LW

**Fig. 5:**
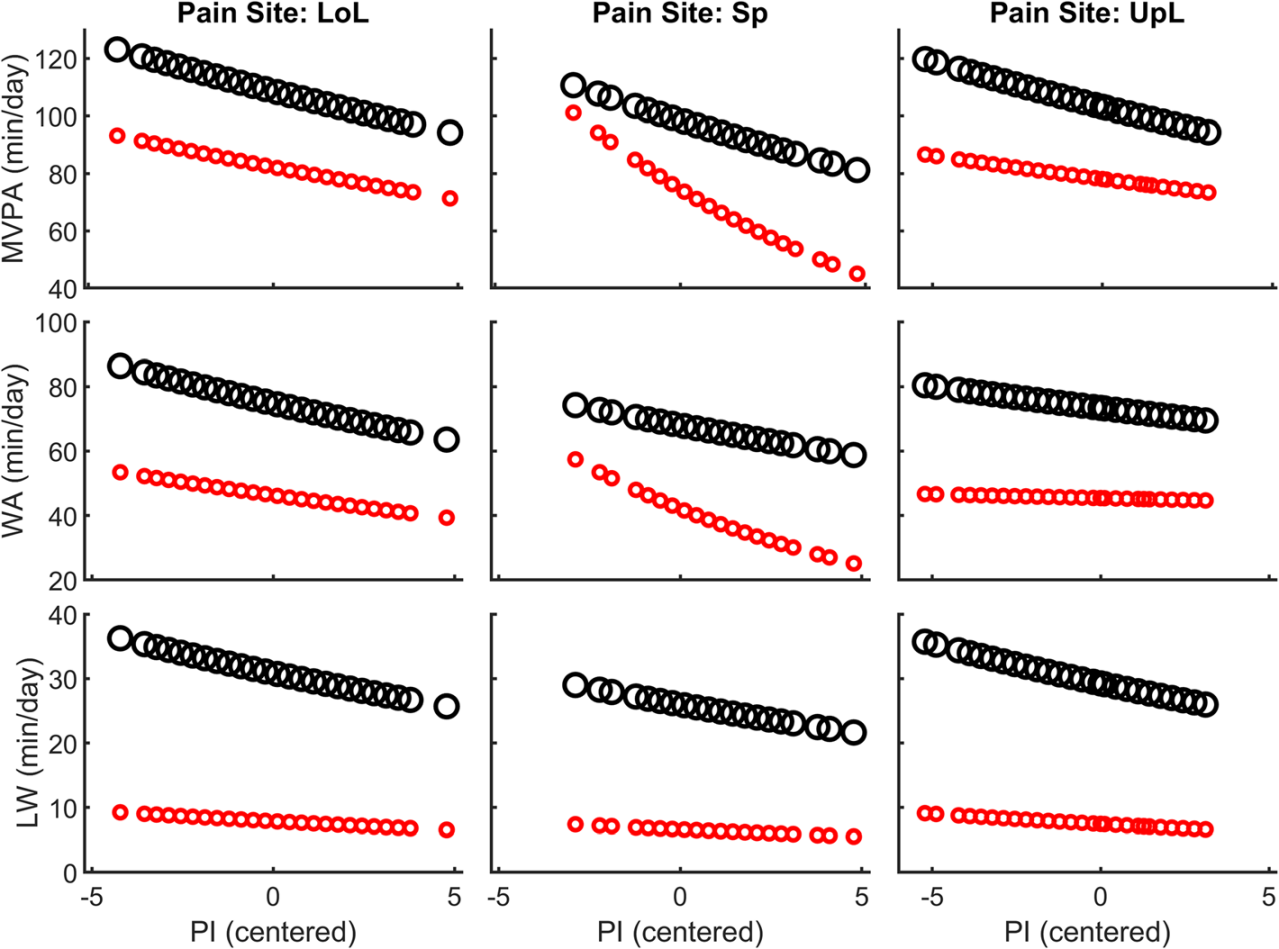
Effect of pain interference (PI) on physical and walking activity split by pain site, as predicted by the generalized linear mixed models (marginal effects). Large black circles are rehabilitation days and small red circles are days off. LoL: lowers limbs. Sp: spine. UpL: upper limbs. MVPA: moderate-to-vigorous physical activity. WA: walking activity. LW: long walk

With models containing more predictors, the day type was still the variable that explained most variance in the dependent variables (Table 4). Regarding LW, the pain site and PI had no effect and did not modulate the effect of rehabilitation. Regarding WA, patients with back pain differed from the other patients in terms of the effect of PI and rehabilitation (triple interaction: pain site x PI x day type). This effect is more clearly highlighted in Fig. 5 (middle panel). Regarding MVPA, PI tended to have a different effect for LoL and Sp patients [one point change in PI score is associated with a 2.9% MVPA decrease for LoL, 10.2% for SP, and 1.9% for UpL (NS)]. Here too, PI modulated rehabilitation effects differently for back-pain patients (significant PI x pain site x day type interaction). That is, the effect of PI during the stay was attenuated: back-pain patients who reported high PI increased more their activity when they were at the clinic than back-pain patients who reported low PI (Fig. 5, middle upper panel).

**Table 4:** Determinants of physical and walking activity

Model 5–7	Coefficient	Confidence interval		% change	Confidence interval	
(*n* = 1360 days)	Estimate	Lower	Upper	exp(coeff.)-1	Lower	Upper
*MVPA* (Model 5)						
(intercept)	**4.409**	4.333	4.486	(82.2)	(76.2)	(88.8)
Day type						
(Days off)						
Rehabilitation days	**0.279**	0.219	0.338	32.1%	24.5%	40.2%
Pain interference (PI)	**−0.030**	− 0.060	0.000	−2.9%	−5.8%	0.0%
Pain site						
(Lower limbs)						
Spine (Sp)	−0.098	− 0.201	0.005	−9.3%	−18.2%	0.5%
Upper limbs (UpL)	−0.052	− 0.137	0.033	−5.1%	−12.8%	3.4%
Interactions						
PI x Sp	**−0.076**	−0.137	− 0.015	−7.3%	− 12.8%	− 1.5%
PI x UpL	0.010	− 0.042	0.062	1.0%	−4.1%	6.4%
Rehab. days x PI x SP	**0.065**	0.013	0.118	6.7%	1.3%	12.5%
Rehab. days x PI x UpL	−0.009	−0.051	0.033	−0.9%	−5.0%	3.4%
*WA* (Model 6)						
(intercept)	**3.835**	3.739	3.931	(46.3)	(42.1)	(51.0)
Day type						
(days off)						
Rehabilitation days	**0.480**	0.406	0.555	61.6%	50.1%	74.2%
Pain interference (PI)	−0.034	−0.071	0.003	−3.3%	−6.9%	0.3%
Pain site						
(Lower limbs)						
Spine (Sp)	−0.095	−0.224	0.033	−9.1%	−20.1%	3.4%
Upper limbs (UpL)	−0.019	−0.125	0.087	−1.9%	−19.3%	9.1%
Interactions						
PI x Sp	−0.074	−0.150	0.003	−7.1%	− 13.9%	0.3%
PI x UpL	0.029	−0.036	0.094	2.9%	−3.5%	9.9%
Rehab. days x PI x SP	**0.077**	0.011	0.143	8.0%	1.1%	15.4%
Rehab. days x PI x UpL	−0.012	−0.065	0.041	−1.2%	−6.3%	4.2%
*LW* (Model 7)						
(intercept)	**2.063**	1.849	2.276	(7.9)	(6.4)	(9.7)
Day type						
(days off)						
Rehabilitation days	**1.366**	1.190	1.542	292.1%	228.7%	367.4%
Pain interference (PI)	−0.038	−0.078	0.002	−3.8%	−7.5%	0.2%
Pain site						
(Lower limbs)						
Spine (Sp)	−0.172	−0.393	0.049	−15.8%	−32.5%	5.0%
Upper limbs (UpL)	−0.053	−0.244	0.137	−5.2%	−21.7%	14.7%

Fixed effects of generalized linear mixed models (Gamma distribution with log link). Full model outputs are shown in Additional file 1: Tables S5-S10. Boldface indicates that the coefficient is significantly different from zero. *MVPA* moderate-to-vigorous physical activity, *WA* walking activity, *LW* long walks (> 1 min)

## Discussion

We studied physical activity levels and walking behaviors in 272 inpatients with chronic pain hospitalized for multidisciplinary rehabilitation. First, the results suggest that measuring days-off during a patient’s stay can serve as a proxy for habitual physical activity. Second, study participants increased their physical activity substantially at the clinic (+ 35%). This increase is mainly explained by more frequent long walks (+ 300%). Finally, self-perception of physical functioning with pain—measured by means of BPI—was associated with activity level, especially in patients with back pain.

Regarding the assessment method, we considered as MVPA all whole-body movements that induced higher accelerations than a slow gait. The classical MVPA definition relies on metabolic equivalent of task (MET), which must be higher than 3 [46]. Therefore, slow walking is categorized as sedentary-to-light physical activity, and only normal and brisk walking are classified as MVPA. The reason for not following this definition was twofold. First, patients suffering chronic pain have a limited capacity to walk fast and a lower preferred walking speed [41, 47–50]. Second, slow walking speeds induce higher energy cost of displacement, and pain may alter walking efficiency [50, 51]; that is, pain patients may expend more energy and may be more rapidly exhausted than healthy counterparts over similar walking distance. Therefore, our definition of MVPA seems more adapted for individuals in pain and with limited walking abilities.

Considering the study’s methodology further, we experienced many missing days due to poor compliance in wearing the accelerometer, especially during weekends. We are confident that we correctly captured average activity patterns, given the large number of observed days and the use of mixed models, which are inherently robust to missing values. However, this issue should be addressed in case of individual assessment of physical activity in future clinical applications, for example by using a device that can be worn 24 h a day. Another methodological issue is that MVPA estimation might be slightly underestimated, because inpatients may practice cycloergometer and strength exercises that are poorly measured with an accelerometer fixed onto the hip.

The first study goal was to explore whether measuring days off during the rehabilitation stay could be a solution assessing habitual physical activity, because it can be logistically complicated to measure patients at home before hospitalization. The results (Fig. 2 and Tables 2 and 3) demonstrate that weekend days during the stay have similar MVPA as days measured before the stay. By measuring physical activity during days off, medical staff can therefore obtain information about patient’s habitual behavior for a better evaluation of functional status.

During days off, study’s participants spent 90% of their waking time in sedentary activities (Fig. 4). WA accounted for 57% of MVPA, i.e., 43 min per day. A substantial inter-individual variability was observed (MVPA CV = 67%, WA CV = 79%). Activity level and walking behavior are indeed labile parameters that strongly depend on occupation and personal habits, along with multiple social and environmental variables [52–55]. Although there is no conclusive evidence that patients with chronic pain are globally less active than healthy people [56, 57], the study of Ryan et al. [53] highlighted that individuals with chronic low back pain walked less than matched controls (9 min/h versus. 13 min / hour on non-work days). For comparison, our results show 3 min/h; this lower value is likely due to the higher severity of symptoms in our sample.

Inpatients substantially increased their physical activity level when staying at our rehabilitation clinic (+ 35%) by adding each day 28 min of MVPA to their habitual level (Fig. 4). Evidence exists that biopsychosocial rehabilitation improves pain and physical function in patients suffering chronic low back pain [58] and other pain conditions [10, 47]. To date, the efficacy of extensive rehabilitation programs has been mostly attributed to the synergy between physical and psychological therapies. Here, we show that an increase of daily physical activity could also contribute to the overall positive outcome of hospitalization for biopsychosocial rehabilitation.

Our results highlight a negative relationship between MVPA and PI (Table 4 and Fig. 5) for patients with LoL and Sp pain. In other words, the patients who reported that pain interfered substantially with their daily activities were effectively less active. The absence of a significant association between PI and MVPA for patients with upper-limb pain is very likely due to the poor recording of upper-limb movements with the accelerometer fixed to the hip. Regarding LoL patients, the strength of association (− 3% MVPA per PI point change) seems relevant: A patient with a score of 8 would be 15% less active than a patient with a score of 3. This relationship is substantially stronger in patients with back pain. For these patients, a five-point difference on the PI scale would correspond to a 50% change in MVPA. Similar results have been described in the literature regarding low back pain, as reported in the meta-analysis of Lin et al. [59]. In line with these studies, our results thus support the hypothesis that a link exists between perceived physical functioning and actual physical activity level.

Study participants were more active during rehabilitation days, mainly through participation in more long walks (Fig. 4). To be more precise, participants averaged 7 min of long walking bouts during days off, and they increased this duration to 29 min during rehabilitation days (+ 22 min). Total WA and MVPA increased accordingly (+ 32 min and + 28 min, respectively). Whereas walking constituted about half of MVPA (57%) during days off, walking became dominant during rehabilitation days (72%). Modifying walking behavior seems therefore a potent mean to make patients more active.

A close examination of the data led us to conclude that the spatial organization of clinic facilities was the main explanation for the WA increase on rehabilitation days. During a typical rehabilitation day, the inpatients had to move between their bedrooms, the therapy facilities, and the restaurant. In other words, the inpatients were obligated to walk for accomplishing their rehabilitation program, even beside their therapies. Furthermore, given the relative smallness of hospital’s bedrooms, inpatients were motivated to walk indoor and outdoor for socializing and enjoying clinic leisure facilities. Given the size of the building, most of paths among therapy rooms and other facilities necessitate 50–150 m walks, which last more than 1 min; this explains the large augmentation of long walks during rehabilitation days (+ 302%).

The obligation of frequent displacements may thus be an important incentive that can push patients to be active through more long walks. In addition to prescribing exercise, adapting the walkability of a patient’s environment, as well as including more walks in a patient’s daily activities, could therefore be used to enhance physical activity. Correspondingly, changing the built environment to increase walking practice and physical activity level of populations is a very active area of research [55, 60]. Besides, it is well established that walking can reduce pain and improve self-reported physical function [24]. However, further investigations are needed to clarify whether unstructured walking practiced in many discontinuous bouts can have the same positive effects as structured walking exercises of longer duration.

The strength of the current study is that it includes a substantial number of patients assessed in a constant environment. The biopsychosocial complexity of the included patients was comparable to the average complexity of the hospitalized patients at the Clinique Romande de Réadaptation. Therefore, the study’s sample was very likely representative of the inpatient population. Furthermore, patients are referred from all the French-speaking counties of Switzerland, including both rural and urban areas. Excepting the underrepresentation of women, the study’s sample is therefore expected to accurately represent the Swiss injured population.

The first limitation of the study is that it took place in a single center. Because we assumed that changes of physical activity were mainly driven by the spatial distribution of the clinic facilities, an identical physical activity enhancement is not expected for other clinics and hospitals. Nevertheless, we are confident that the main finding of the study—that patients suffering from chronic pain can be made more active through walking incentives—is valid for other settings. Second, we focused on the immediate effects of extensive rehabilitation on physical activity levels. The potential carry-over effects after discharge should be further investigated.

## Conclusions

Although there is compelling evidence that maintaining a high level of physical activity has positive effects, many healthcare professionals hesitate to prescribe exercise to treat chronic pain [61, 62]. The erroneous belief that excessive movements may worsen pain is widespread [34, 63]. Fear-avoidance beliefs and behaviors exist both in patients and caregivers [34, 64], who may be inclined to consider patients as unable to be more physically active. In contrast, the present study shows that physical activity levels can be enhanced via appropriate incentives, even in patients reporting a high degree of impairment in their physical functioning due to pain. This finding should further motivate healthcare professionals to promote physical activity to their patients suffering from chronic pain. In practice, adding more walking in a patient’s daily activities could be an effective strategy.

## Additional files

Additional file 1: Supplementary material. The document shows additional figures (data distribution, residual plots) and tables (detailed statistics). (PDF 1.67 MB)

Additional file 2: Excel spreadsheet. Raw data. The spreadsheet contains study’s raw data. (XLSX 141 kb)

## Abbreviations

CV: Coefficient of variation
LoL: Lower limbs
LW: Long walks
MVPA: Moderate-to-vigorous physical activity
NS: Not significant
PI: Pain interference
Sp: Spine
UpL: Upper limbs
WA: Walking activity
