# Supplementary material for: Influencing walking behavior can increase the physical activity of patients with chronic pain hospitalized for multidisciplinary rehabilitation: an observational study
Source: BMC Musculoskelet Disord. 2019 May 4;20:188. doi: 10.1186/s12891-019-2561-9 (PMC6500043; doi:10.1186/s12891-019-2561-9)
Supplement: Supplementary file 1 — Supplementary material. The document shows additional figures (data distribution, residual plots) and tables (detailed statistics). (PDF 1.67 MB) [file 12891_2019_2561_MOESM1_ESM.pdf]

# Influencing walking behavior can increase the physical activity of patients with chronic pain hospitalized for multidisciplinary rehabilitation: an observational study

https://doi.org/10.1186/s12891-019-2561-9

Philippe Terrier[a,b,c], Caroline Praz[b,c], Joane Le Carré[b,c], Philippe Vuistiner[b,c], Bertrand Léger[b,c], François Luthi[b,c].

[a] Haute Ecole Arc Santé, HES-SO University of Applied Sciences and Arts Western Swizerland, Neuchâtel, Switzerland
[b] IRR, Institute for Research in Rehabilitation, Sion, Switzerland
[c] Clinique romande de réadaptation SUVACare, Sion, Switzerland

## Supplementary Figures and Tables

**MODEL 1 (Figure S1 & Table S1)** ........ p. 2

**MODEL 2 (Figure S2 & Table S2)** ........ p. 3

**MODEL 3 (Figure S3 & Table S3)** ........ p. 4

**MODEL 4 (Figure S4 & Table S4)** ........ p. 5

**MODEL 5 (Table S5 & Table S6)** ........ p. 6

**MODEL 6 (Table S7 & Table S8)** ........ p. 7

**MODEL 7 (Table S9 & Table S10)** ........ p. 8

**RESIDUALS (Figure S5)** ........ p. 9

# Model 1: Moderate-to-vigorous physical activity at home and at the rehabilitation clinic

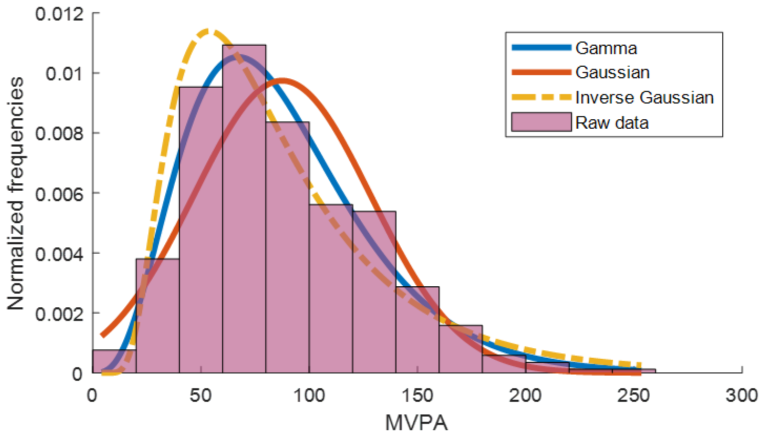


***Figure S1. MVPA Histogram.*** *Normalized frequencies as a function of MVPA and best-fit curves for Gamma, Gaussian and Inverse Gaussian distributions. N= 855 days.*

**Table S1. Matlab output of Model 1.**

```
mdl=fitglme(data,'MVPA ~ 1 + Day_type +
(Day_type|Subject)','Distribution','Gamma','link','log','FitMethod','REMPL');


Generalized linear mixed-effects model fit by PL

Model information:
    Number of observations              855
    Fixed effects coefficients            4
    Random effects coefficients         372
    Covariance parameters                11
    Distribution                     Gamma
    Link                             Log
    FitMethod                        REMPL

Formula:
    MVPA ~ 1 + Day_type + (1 + Day_type | Subject)

Model fit statistics:
    AIC       BIC       LogLikelihood    Deviance
    816.73    887.93    -393.37          786.73

Fixed effects coefficients (95% CIs):
    Name                     Estimate    SE          tStat     DF     pValue        Lower       Upper
    '(Intercept)'              4.174     0.058212    71.702    851             0      4.0597     4.2882
    'Day_type_2'             0.15531     0.038882    3.9945    851     7.044e-05    0.078997    0.23163
    'Day_type_3'             0.12437     0.048288    2.5756    851      0.010176    0.029591    0.21914
    'Day_type_4'             0.38848     0.058894    6.5963    851     7.401e-11     0.27289    0.50408

Random effects covariance parameters:
Group: Subject (93 Levels)
    Name1                    Name2                    Type            Estimate
    '(Intercept)'            '(Intercept)'            'std'             0.48213
    'Day_type_2'             '(Intercept)'            'corr'           -0.64506
    'Day_type_3'             '(Intercept)'            'corr'           -0.73858
    'Day_type_4'             '(Intercept)'            'corr'           -0.81103
    'Day_type_2'             'Day_type_2'             'std'             0.18637
    'Day_type_3'             'Day_type_2'             'corr'          -0.030467
    'Day_type_4'             'Day_type_2'             'corr'            0.21964
    'Day_type_3'             'Day_type_3'             'std'             0.18957
    'Day_type_4'             'Day_type_3'             'corr'            0.81085
    'Day_type_4'             'Day_type_4'             'std'             0.44055

Group: Error
    Name                          Estimate
    'sqrt(Dispersion)'            0.30944
```

# Model 2: Univariable GLMM for day type effects on moderate-to-vigorous physical activity (MVPA)

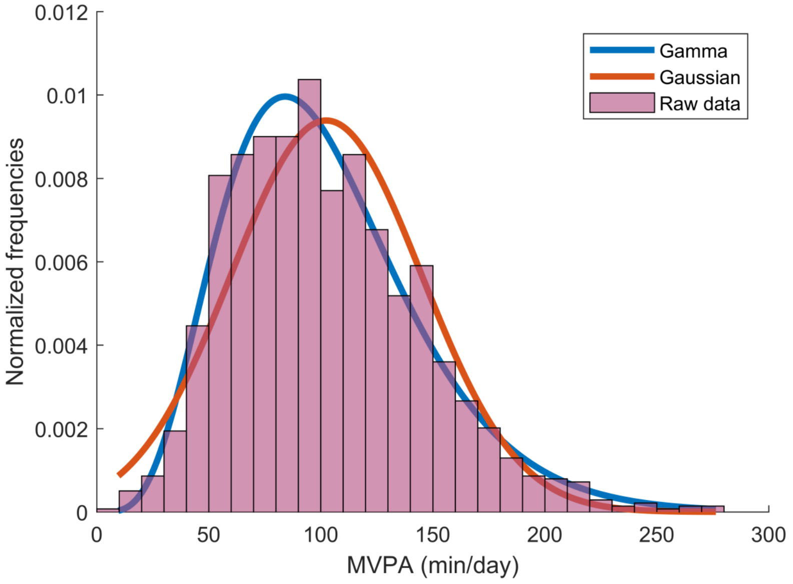


***Figure S2. MVPA Histogram.*** *Normalized frequencies as a function of MVPA and best-fit curves for Gamma and Gaussian distributions. N= 1388 days.*

**Table S2. Matlab output of Model 2.**

```
mdl =fitglme(data,'MVPA ~ 1 + Day_Type +
(Day_Type|Subject)','Distribution','Gamma','link','log','FitMethod','REMPL');

Generalized linear mixed-effects model fit by PL

Model information:
    Number of observations             1388
    Fixed effects coefficients            2
    Random effects coefficients         544
    Covariance parameters                 4
    Distribution                      Gamma
    Link                              Log
    FitMethod                         REMPL

Formula:
    MVPA ~ 1 + Day_Type + (1 + Day_Type | Subject)

Model fit statistics:
    AIC       BIC       LogLikelihood    Deviance
    1054.5    1085.9    -521.25          1042.5

Fixed effects coefficients (95% CIs):
    Name                 Estimate    SE          tStat     DF      pValue         Lower      Upper
    '(Intercept)'          4.346     0.028928    150.23    1386             0      4.2893     4.4028
    'Day_Type_1'         0.29655     0.028327    10.469    1386    9.7834e-25    0.24098    0.35212

Random effects covariance parameters:
Group: Subject (272 Levels)
    Name1                Name2                Type           Estimate
    '(Intercept)'        '(Intercept)'        'std'           0.38555
    'Day_Type_1'         '(Intercept)'        'corr'         -0.68456
    'Day_Type_1'         'Day_Type_1'         'std'           0.34486

Group: Error
    Name                      Estimate
    'sqrt(Dispersion)'        0.26912
```

# Model 3: Univariable GLMM for day type effects on walking activity (WA)

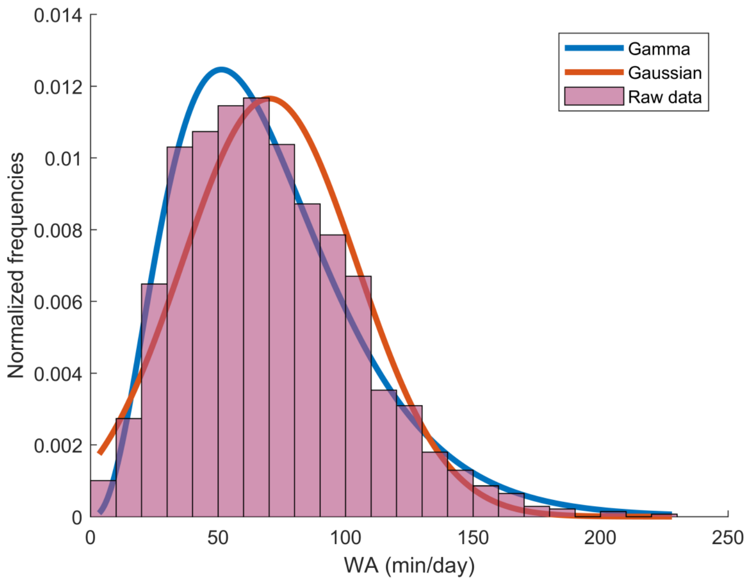


***Figure S3. WA Histogram.*** *Normalized frequencies as a function of WA and best-fit curves for Gamma and Gaussian distributions. N= 1388 days.*

**Table S3. Matlab output of Model 3.**

```
mdl =fitglme(data,'WA~ 1 + Day_Type +
(Day_Type|Subject)','Distribution','Gamma','link','log','FitMethod','REMPL');

Generalized linear mixed-effects model fit by PL

Model information:
    Number of observations             1388
    Fixed effects coefficients            2
    Random effects coefficients         544
    Covariance parameters                 4
    Distribution                    Gamma
    Link                            Log
    FitMethod                       REMPL

Formula:
    WA ~ 1 + Day_Type + (1 + Day_Type | Subject)

Model fit statistics:
    AIC       BIC       LogLikelihood    Deviance
    1528.5    1559.9    -758.23          1516.5

Fixed effects coefficients (95% CIs):
    Name                   Estimate     SE           tStat      DF       pValue           Lower      Upper
    '(Intercept)'           3.7781      0.035947      105.1     1386              0       3.7076     3.8487
    'Day_Type_1'           0.50425      0.035709     14.121     1386     2.1052e-42       0.4342     0.5743

Random effects covariance parameters:
Group: Subject (272 Levels)
    Name1                  Name2                   Type          Estimate
    '(Intercept)'          '(Intercept)'           'std'          0.48745
    'Day_Type_1'           '(Intercept)'           'corr'        -0.70506
    'Day_Type_1'           'Day_Type_1'            'std'          0.45291

Group: Error
    Name                        Estimate
    'sqrt(Dispersion)'          0.3119
```

# Model 4: Univariable GLMM for day type effects on long walks (LW)

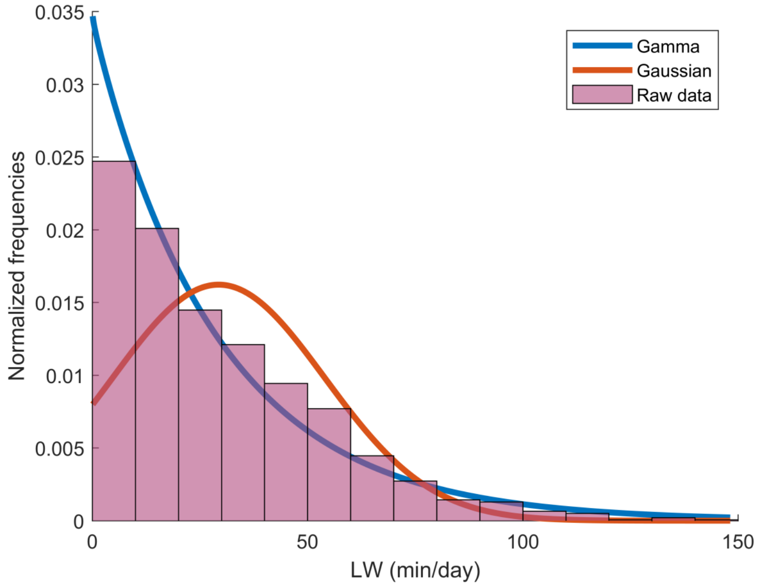


***Figure S4. LW Histogram.*** *Normalized frequencies as a function of LW and best-fit curves for Gamma and Gaussian distributions. N= 1388 days.*

**Table S4. Matlab output of Model 4.**

```
mdl =fitglme(data,'LW~ 1 + Day_Type +
(Day_Type|Subject)','Distribution','Gamma','link','log','FitMethod','REMPL');

Generalized linear mixed-effects model fit by PL

Model information:
    Number of observations             1388
    Fixed effects coefficients            2
    Random effects coefficients         544
    Covariance parameters                 4
    Distribution                    Gamma
    Link                            Log
    FitMethod                       REMPL

Formula:
    LW ~ 1 + Day_Type + (1 + Day_Type | Subject)

Model fit statistics:
    AIC       BIC       LogLikelihood    Deviance
    3329.2    3360.6    -1658.6          3317.2

Fixed effects coefficients (95% CIs):
    Name                 Estimate    SE          tStat     DF      pValue        Lower     Upper
    '(Intercept)'        1.9786      0.092186    21.463    1386    1.8507e-88    1.7978    2.1594
    'Day_Type_1'         1.3917      0.090186    15.431    1386    1.0657e-49    1.2148    1.5686

Random effects covariance parameters:
Group: Subject (272 Levels)
    Name1                Name2                Type          Estimate
    '(Intercept)'        '(Intercept)'        'std'           1.3207
    'Day_Type_1'         '(Intercept)'        'corr'        -0.88808
    'Day_Type_1'         'Day_Type_1'         'std'           1.2464

Group: Error
    Name                      Estimate
    'sqrt(Dispersion)'        0.56993
```

# Model 5: Multivariable GLMM for moderate-to-vigorous physical activity (MVPA)

**Table S5. Matlab ANOVA analysis of Model 5**

```
mdl = fitglme(data,'MVPA ~ 1 + PI * Site * Day_Type +
(Day_Type|Subject)','Distribution','Gamma','link','log','FitMethod','MPL');

anova(mdl)

ANOVA marginal tests: DFMethod = 'residual'

    Term                      FStat       DF1    DF2     pValue
    '(Intercept)'               8123.5    1      1348             0
    'Day_Type'                  43.302    1      1348    6.6972e-11
    'PI'                         0.651    1      1348        0.4199
    'Site'                    0.078317    2      1348       0.92468
    'Day_Type:PI'              0.41375    1      1348       0.52018
    'Day_Type:Site'             1.2733    2      1348       0.28024
    'PI:Site'                   4.6021    2      1348      0.010189
    'Day_Type:PI:Site'          4.0144    2      1348      0.018271
```

**Table S6. Matlab output of Model 5**

```
mdl = fitglme(data,'MVPA ~ 1 + Day_Type + PI * Site + Day_Type:PI:Site +
(Day_Type|Subject)','Distribution','Gamma','link','log','FitMethod','REMPL');


Generalized linear mixed-effects model fit by PL

Model information:
    Number of observations             1360
    Fixed effects coefficients            9
    Random effects coefficients         534
    Covariance parameters                 4
    Distribution                    Gamma
    Link                            Log
    FitMethod                       REMPL

Formula:
    MVPA ~ 1 + Day_Type + PI*Site + Day_Type:PI:Site + (1 + Day_Type | Subject)

Model fit statistics:
    AIC       BIC       LogLikelihood    Deviance
    1034.5    1102.2    -504.26          1008.5

Fixed effects coefficients (95% CIs):
    Name                      Estimate       SE          tStat      DF      pValue         Lower          Upper
    '(Intercept)'              4.4094      0.039106     112.76     1351            0        4.3327          4.4861
    'Day_Type_1'               0.2786      0.030132      9.246     1351    8.8002e-20      0.21949         0.33771
    'PI'                     -0.029748     0.01524     -1.9519     1351      0.051153    -0.059644      0.00014923
    'Site_2'                 -0.09783      0.052583    -1.8605     1351      0.063032     -0.20098       0.0053226
    'Site_3'                 -0.051856     0.043377     -1.1955    1351       0.23212     -0.13695        0.033238
    'PI:Site_2'              -0.075762     0.031161    -2.4313     1351      0.015174     -0.13689       -0.014633
    'PI:Site_3'               0.0098085     0.0264      0.37153    1351        0.7103    -0.041982        0.061599
    'Day_Type_1:PI:Site_2' 0.065155     0.02675       2.4357     1351      0.014991     0.012679         0.11763
    'Day_Type_1:PI:Site_3'-0.0087286    0.02138      -0.40826    1351       0.68314     -0.05067        0.033213

Random effects covariance parameters:
Group: Subject (267 Levels)
    Name1               Name2               Type          Estimate
    '(Intercept)'       '(Intercept)'       'std'         0.37114
    'Day_Type_1'        '(Intercept)'       'corr'        -0.6916
    'Day_Type_1'        'Day_Type_1'        'std'         0.34786

Group: Error
    Name                      Estimate
    'sqrt(Dispersion)'        0.26535
```

# Model 6: Multivariable GLMM for walking activity (WA)

**Table S7. Matlab ANOVA analysis of Model 6**

```
mdl = fitglme(data,'WA ~ 1 + PI * Site * Day_Type +
(Day_Type|Subject)','Distribution','Gamma','link','log','FitMethod','MPL');

anova(mdl)

ANOVA marginal tests: DFMethod = 'residual'

    Term                       FStat      DF1     DF2      pValue
    '(Intercept)'               3864.1    1       1348             0
    'Day_Type'                  78.925    1       1348    2.0177e-18
    'PI'                       0.66817    1       1348       0.41383
    'Site'                     0.11266    2       1348       0.89346
    'Day_Type:PI'              0.22924    1       1348       0.63216
    'Day_Type:Site'             1.7164    2       1348        0.1801
    'PI:Site'                   3.8504    2       1348      0.021505
    'Day_Type:PI:Site'          3.7847    2       1348      0.022958
```

**Table S8. Matlab ouput of Model 6**

```
mdl = fitglme(data,'WA ~ 1 + Day_Type + PI * Site + Day_Type:PI:Site +
(Day_Type|Subject)','Distribution','Gamma','link','log','FitMethod','REMPL');


Generalized linear mixed-effects model fit by PL

Model information:
    Number of observations             1360
    Fixed effects coefficients            9
    Random effects coefficients         534
    Covariance parameters                 4
    Distribution                      Gamma
    Link                              Log
    FitMethod                         REMPL

Formula:
    WA ~ 1 + Day_Type + PI*Site + Day_Type:PI:Site + (1 + Day_Type | Subject)

Model fit statistics:
    AIC       BIC       LogLikelihood    Deviance
    1517.7    1585.4    -745.85          1491.7

Fixed effects coefficients (95% CIs):
    Name                        Estimate     SE           tStat        DF      pValue        Lower         Upper
    '(Intercept)'                 3.8349      0.04891       78.409     1351             0         3.739        3.9309
    'Day_Type_1'                 0.48015     0.037926        12.66     1351     8.526e-35       0.40575       0.55455
    'PI'                       -0.034033     0.018993      -1.7919     1351      0.073381     -0.071293     0.0032264
    'Site_2'                   -0.095419     0.065431      -1.4583     1351       0.14499      -0.22378      0.032938
    'Site_3'                   -0.019085     0.054044     -0.35314     1351       0.72404       -0.1251      0.086934
    'PI:Site_2'                -0.073756     0.038998      -1.8913     1351      0.058798      -0.15026     0.0027463
    'PI:Site_3'                 0.028772     0.033037      0.87093     1351       0.38395     -0.036036      0.093581
    'Day_Type_1:PI:Site_2'      0.077269     0.033639        2.297     1351       0.02177      0.011279       0.14326
    'Day_Type_1:PI:Site_3'     -0.012245     0.026914     -0.45498     1351        0.6492     -0.065043      0.040552

Random effects covariance parameters:
Group: Subject (267 Levels)
    Name1                 Name2                 Type           Estimate
    '(Intercept)'         '(Intercept)'         'std'          0.47501
    'Day_Type_1'          '(Intercept)'         'corr'         -0.7028
    'Day_Type_1'          'Day_Type_1'          'std'          0.45362

Group: Error
    Name                       Estimate
    'sqrt(Dispersion)'         0.30959
```

# Model 7: Multivariable GLMM for long walks (LW)

**Table S9. Matlab ANOVA analysis of Model 7**

```
mdl = fitglme(data,'LW ~ 1 + PI * Site * Day_Type +
(Day_Type|Subject)','Distribution','Gamma','link','log','FitMethod','MPL');

anova(mdl)

ANOVA marginal tests: DFMethod = 'residual'

    Term                        FStat      DF1     DF2      pValue
    '(Intercept)'                157.23    1       1348     3.3929e-34
    'Day_Type'                   91.817    1       1348     4.3849e-21
    'PI'                        0.45594    1       1348        0.49964
    'Site'                      0.43166    2       1348        0.64952
    'Day_Type:PI'               0.04171    1       1348         0.8382
    'Day_Type:Site'              1.7554    2       1348        0.17323
    'PI:Site'                    1.8851    2       1348        0.15221
    'Day_Type:PI:Site'            2.126    2       1348        0.11971
```

**Table S10. Matlab output of Model 7**

```
mdl = fitglme(data,'LW ~ 1 + Day_Type + PI + Site +
(Day_Type|Subject)','Distribution','Gamma','link','log','FitMethod','REMPL');

Generalized linear mixed-effects model fit by PL

Model information:
    Number of observations              1360
    Fixed effects coefficients             5
    Random effects coefficients          534
    Covariance parameters                  4
    Distribution                       Gamma
    Link                               Log
    FitMethod                          REMPL

Formula:
    LW ~ 1 + Day_Type + PI + Site + (1 + Day_Type | Subject)

Model fit statistics:
    AIC        BIC        LogLikelihood    Deviance
    3261.5     3308.4     -1621.8          3243.5

Fixed effects coefficients (95% CIs):
    Name                    Estimate     SE          tStat      DF      pValue         Lower         Upper
    '(Intercept)'             2.0627     0.10886     18.949     1355    3.2422e-71        1.8492        2.2763
    'Day_Type_1'              1.3663     0.08965      15.24     1355    1.5862e-48        1.1904        1.5422
    'PI'                   -0.038271     0.02033    -1.8825     1355      0.059983     -0.078153     0.0016106
    'Site_2'                -0.17206     0.11282    -1.5251     1355       0.12746      -0.39338      0.049257
    'Site_3'               -0.053089    0.097145    -0.5465     1355       0.58481      -0.24366       0.13748

Random effects covariance parameters:
Group: Subject (267 Levels)
    Name1                   Name2                   Type           Estimate
    '(Intercept)'           '(Intercept)'           'std'            1.3062
    'Day_Type_1'            '(Intercept)'           'corr'         -0.88596
    'Day_Type_1'            'Day_Type_1'            'std'            1.2217

Group: Error
    Name                        Estimate
    'sqrt(Dispersion)'          0.56787
```

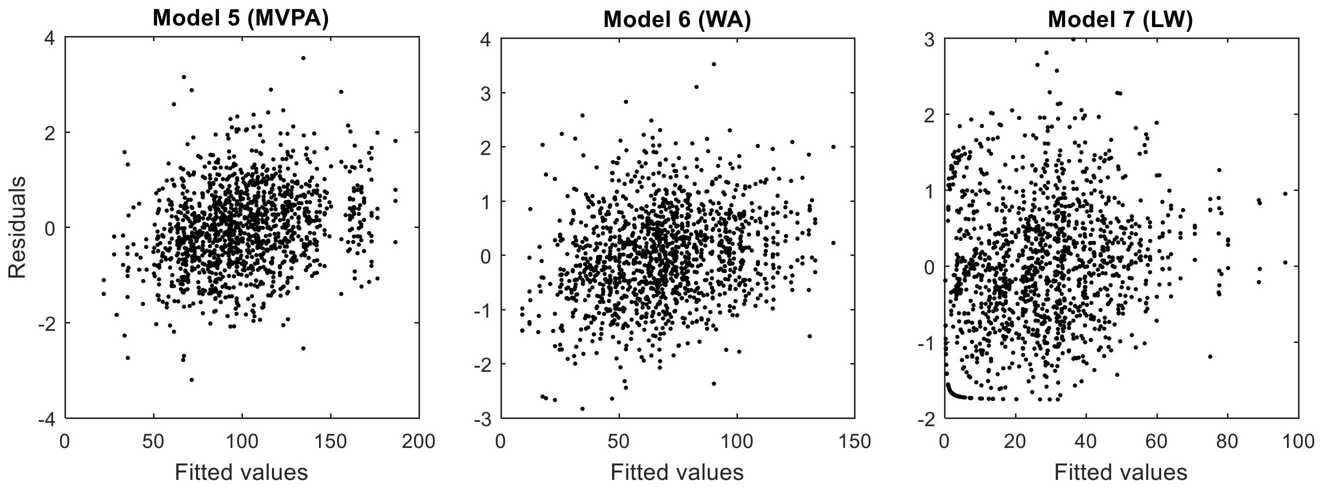


**Fig. S5. Pearson's residuals of the multivariable models. Contributions from both fixed effects and random effects (conditional).**
